# Characterisation of a secretory serine protease inhibitor (SjB6) from *Schistosoma japonicum*

**DOI:** 10.1186/1756-3305-7-330

**Published:** 2014-07-14

**Authors:** Adebayo J Molehin, Geoffrey N Gobert, Patrick Driguez, Donald P McManus

**Affiliations:** 1 Molecular Parasitology Laboratory, QIMR Berghofer Medical Research Institute, 300 Herston Road, Herston 4006, Australia; 2 School of Population Health, The University of Queensland, 300 Herston Road, Herston, Brisbane 4006, Australia

**Keywords:** *Schistosoma japonicum*, Serpin, SjB6, Host-parasite interaction, Structural analysis

## Abstract

**Background:**

Proteins belonging to the serine protease inhibitor (serpin) superfamily play essential physiological roles in many organisms. In pathogens, serpins are thought to have evolved specifically to limit host immune responses by interfering with the host immune-stimulatory signals. Serpins are less well characterised in parasitic helminths, although some are thought to be involved in mechanisms associated with host immune modulation. In this study, we cloned and partially characterised a secretory serpin from *Schistosoma japonicum* termed SjB6, these findings provide the basis for possible functional roles.

**Methods:**

*SjB6* gene was identified through database mining of our previously published microarray data, cloned and detailed sequence and structural analysis and comparative modelling carried out using various bioinformatics and proteomics tools. Gene transcriptional profiling was determined by real-time PCR and the expression of native protein determined by immunoblotting. An immunological profile of the recombinant protein produced in insect cells was determined by ELISA.

**Results:**

*SjB6* contains an open reading frame of 1160 base pairs that encodes a protein of 387 amino acid residues. Detailed sequence analysis, comparative modelling and structural-based alignment revealed that *SjB6* contains the essential structural motifs and consensus secondary structures typical of inhibitory serpins. The presence of an N-terminal signal sequence indicated that SjB6 is a secretory protein. Real-time data indicated that *SjB6* is expressed exclusively in the intra-mammalian stage of the parasite life cycle with its highest expression levels in the egg stage (p < 0.0001). The native protein is approximately 60 kDa in size and recombinant SjB6 (rSjB6) was recognised strongly by sera from rats experimentally infected with *S. japonicum*.

**Conclusions:**

The significantly high expression of *SjB6* in schistosome eggs, when compared to other life cycle stages, suggests a possible association with disease pathology, while the strong reactivity of sera from experimentally infected rats against rSjB6 suggests that native SjB6 is released into host tissue and induces an immune response. This study presents a comprehensive demonstration of sequence and structural-based analysis of a secretory serpin from a trematode and suggests SjB6 may be associated with important functional roles in *S. japonicum,* particularly in parasite modulation of the host microenvironment.

## Background

Serine protease inhibitors (serpins) are members of an important superfamily of structurally-related proteins found in many organisms including viruses, bacteria, animals and plants [1-4]. Serpins participate in many important physiological processes such as signalling cascades [5], blood coagulation [6,7], fibrinolysis [8], inflammation and activation of the complement system [9,10]. Furthermore, these inhibitors also play key roles in host immune modulation by pathogens, and hence the suggestion that pathogen serpins may have evolved for the singular purpose of limiting host immune activation by interfering with host immunomodulatory signals [11,12]. In parasitic helminths, these inhibitors appear to perform similar roles [13].

*Schistosoma japonicum* is a zoonotic trematode that infects humans and many animals causing Asiatic schistosomiasis. Despite considerable control measures, high re-infection rates and the potential for the development of drug resistance, necessitate the development of an effective anti-schistosomal vaccine, which would significantly decrease the schistosomiasis-induced morbidity and mortality in endemic areas. Despite significant research regarding the biology and immunology of schistosomiasis, much is still unknown regarding the mechanisms associated with schistosomes evading the host immune responses or the involvement of serpins in these processes*.* In particular, there are limited reports to date presenting the functional characterisation of serpins in the Asiatic schistosomes or their possible role in host-parasite interactions.

Through the analysis of the transcriptional changes occurring during the *S. japonicum* life cycle [14] and cross-referencing with the *S. japonicum* genome database [15], we are able to report here the identification and cloning of a full-length cDNA sequence, termed *SjB6* [GenBank: CAX69453.1] encoding a secretory *S. japonicum* serpin. According to MEROPS classification of protease inhibitors [16], SjB6 (MEROPS Accession: ME179730) is a member of inhibitor family 14 (Clan ID). We also present structure-to-function bioinformatics analysis of the SjB6 polypeptide, its production as a recombinant protein and its characterisation.

## Methods

### Ethics statement

All work was conducted with the approval of the QIMR Berghofer Medical Research Institute Animal Ethics Committee (Project number P288).

### Identification of *S. japonicum* serpin

Source sequence encoding *S. japonicum SjB6* [GenBank: CAX69453.1] was found using BLAST (Basic Local Alignment and Search Tool) [17] with the BLASTP and BLASTN algorithms against *S. japonicum* Gene Index (SjGI) available at DFCI (http://compbio.dfci.harvard.edu/tgi/) and a *S. japonicum* gene expression database created by our research group [14]. Validation of sequence accuracy was carried out by inspecting and confirming the presence of start and stop codons, the expected amino acid length range for serpins (350–450 amino acids of translated protein sequence) [1,18], and the presence of two amino acids motifs described as highly conserved for serpins: NAVYFKG and DVNEEG [19,20].

### Bioinformatics analyses

The *SjB6* coding sequence was compared to known entries in GenBank using the BLASTp program. To gain insight on probable functionality, the deduced SjB6 amino acid sequence was scanned against amino acid motif entries, ScanProsite, THMMM, PROSITE and SignalP servers (ExPASY Bioinformatics Resource Server; http://www.expasy.org/proteomics). The reactive centre loop (RCL) of SjB6 was determined based on the consensus 20/21 residue peptide “p17 [E]-p16 [E/K/R]-p15 [G]-p14 [T/S]-p13 [X]-P12-9 [AGS]-p8-1 [X]-p1’ – 4’” [4,21]. The putative scissile bond (P1 – P1’) and the P1 residue were predicted based on the conserved features that there are generally 17 amino residues (P17 to P1) between the start of the hinge region of the RCL and the scissile bond [1]. Sequence alignment was performed using the MUSCLE algorithm [22,23] in the MEGA 6.0 program [24]. Putative N-glycosylation sites were identified using the NetNGly1.0 server (Gupta *et al*., unpublished, http://cbs.dtu.dk/services/NetNGlyc/). Theoretical molecular weight and isoelectric points of the mature serpin protein were calculated using the ExPASy Compute pI/Mw tool [25].

### Structural based sequence alignment and comparative modelling

The SjB6 amino acid sequence was subjected to structural based alignment using STRAP [26] and the MUSCLE algorithm [22]. The tertiary structures of SjB6 and other parasitic helminth serpins were predicted using the Phyre^2^ program [27]. QMEAN was the method used to estimate model reliability and predict quality [28,29]. The predicted structures were aligned and viewed using DeepView-SWISS PdbViewer v4.1 [30]. Illustrations of the 3D structures were generated using DeepView-SWISS PdbViewer.

### Phylogenetic analysis

Multiple amino acid sequence alignments of SjB6 and some other parasite serpins retrieved from GenBank (GenBank accession numbers in Table 1) were created using the MUSCLE algorithm [22] in an open source Phylogeny.fr program [31]. The phylogenetic analyses were performed using default setting of the “One Click mode” in Phylogeny.fr program [31] with Gblocks for automatic curation [32], PhyML for tree building [33] and TreeDyn for tree drawing [34].

**Table 1:** **Key characteristics of the serpin *SjB6 *** **sequence**

	**Amino acid sequence**	**Amino acid position**
**Serpin motif**	DEEGAV	329 – 334
**Reactive Central Loop**	AASASATVMYMCSAIRSHQPVPE	337 – 357
**Serpin signature**	FRIDHPFFISI	358 – 368

### Parasite materials

A Chinese field isolate (Anhui strain) of *S. japonicum* was maintained in *Oncomelania hupensis hupensis* snails and in BALB/c mice (Animal Resource Centre, Western Australia) at QIMR Berghofer Medical Research Institute. Adult worms were recovered by perfusion of infected mice using sodium citrate buffer (0.15 M sodium chloride/ 0.05 M sodium citrate). Soluble worm antigen products (SWAP) were obtained from homogenised adult worm pairs following centrifugation. Eggs were obtained from infected mouse livers and miracidia hatched from isolated eggs as described [35]. The production of cercariae [36] and schistosomula, obtained by mechanical transformation of cercariae [37], followed published procedures. All parasite stages were stored in liquid nitrogen until needed or stored in RNA*later* (Ambion) at 4°C until total RNA extraction.

### Total RNA extraction, cDNA synthesis and real-time PCR

Total RNA was extracted from isolated *S. japonicum* life cycle stages using Trizol reagent (Life Technologies) and an RNeasy Mini kit (Qiagen). Total RNA quantity was measured using Nanodrop-1000 (Nanodrop Technologies) and quality assessed using an Agilent Bioanalyzer (Agilent Technologies). cDNA was synthesised from total RNA obtained using a Quantitect Reverse Transcription Kit (Qiagen) according to the manufacturer’s instructions. Real-time PCR for *SjB6* was performed using 2.5 ng of cDNAs as templates with the SYBR Green PCR Master Mix (Applied Biosystems). Primers were designed from cDNA sequences using Primer3 (http://primer3.wi.mit.edu/). The forward primer was 5’- TTG ACC AGT TTA CCA CAC CTA CA– 3’ and the reverse primer was 5’- AGA CAG CAA TGA AGA GAT TCC AC – 3’. NADH-ubiquinone reductase (NADH-UR) was used as house-keeping gene [14]. The cycling conditions were: 95°C for 10 min; 39 cycles at 95°C for 30 s at 58°C for 30 s and 72°C for 30 s. All reactions were carried out in four biological replicates. Rotor-Gene 6000 series software and GraphPad Prism software were used to analyse the results.

### Cloning of the *SjB6* gene and sequence analysis

The full-length coding sequence of *SjB6* was amplified from adult worm cDNA by PCR using oligonucleotide primers flanking the open reading frame of the gene designed using Amplify 3 (http://engels.genetics.wisc.edu/amplify/). The forward primer was 5’- CGT ATA CAT TTC TTA CAT CTA TGC GGA TTC GCA TCA CCA TCA CCA TCA CGT TCT TTG CGG TAG TGA TAA TAA TAC GAA AGC T-3’ and the reverse primer was 5’- GTT AGT GGT GGT GGT GGT GGT GTT ATT CAT TCA TTG GTG CTA CAA CAT GTC CTA GA-3’. The amplification reaction was carried out using a thermal cycling profile of 95°C for 2 min; 30 cycles at 95°C for 20 s, 60°C for 20 s, 70°C for 20 s and a final extension for 2 min at 72°C. The PCR product was analysed on a 0.8% (w/v) agarose gel, gel-purified and co-transformed with linearised p-BAC-1 transfer vector into *Escherichia coli* OmniMAX competent cells (Invitrogen). Positive clones were screened for the presence of plasmid with the appropriate insert. The nucleotide sequence of the insert was determined by automated sequencing.

### Generation of recombinant pBAC-1/*SjB6* plasmid DNA

Standard insect cell culture techniques were used as previously described [38,39]. Sf9 cells seeded at 6 × 10^5^ cells/mL in Sf900II medium were plated into a 24-deep well plate (Invitrogen) and transfected with a mixture of 100 ng recombinant pBAC-1/*SjB6* plasmid DNA and 20 ng flashBAC DNA [40] using Cellfectin (Invitrogen) according to the manufacturer’s instructions. Cells were then incubated at 28°C for 5 hr and the supernatant removed afterwards. Media supplemented with Gibco antibiotics-antimycotics (100X) solution (Life Technologies) was added and the cells incubated at 28°C for a further 7 days. Cells were pelleted (500 × *g*, 5 mins) and the supernatant containing P1 virus was stored at 4°C until needed. Cell growth, average cell size and viability were monitored using Countess Automated Cell Counter (Life Technologies).

### Virus amplification and expression of rSjB6

Sf9 cells seeded at 2 × 10^6^ cells/ml were infected with P1 virus and incubated at 28°C at 250 rpm for 4 days in a humidified incubator. At 96 h post-infection, the cells were pelleted and the supernatant containing P2 virus was harvested and stored at 4°C. For the expression of rSjB6, High-Five (Hi-5) cell culture seeded at 1.5 × 10^6^ cells/ml was infected with the P3 virus and incubated at 28°C at 120 rpm for 48 h in a humidified incubator. At 48 h post-infection, the supernatant was collected by centrifugation, filtered through 0.45 μm filter and affinity purified.

### Affinity purification followed by size exclusion chromatography

**Figure 1:**
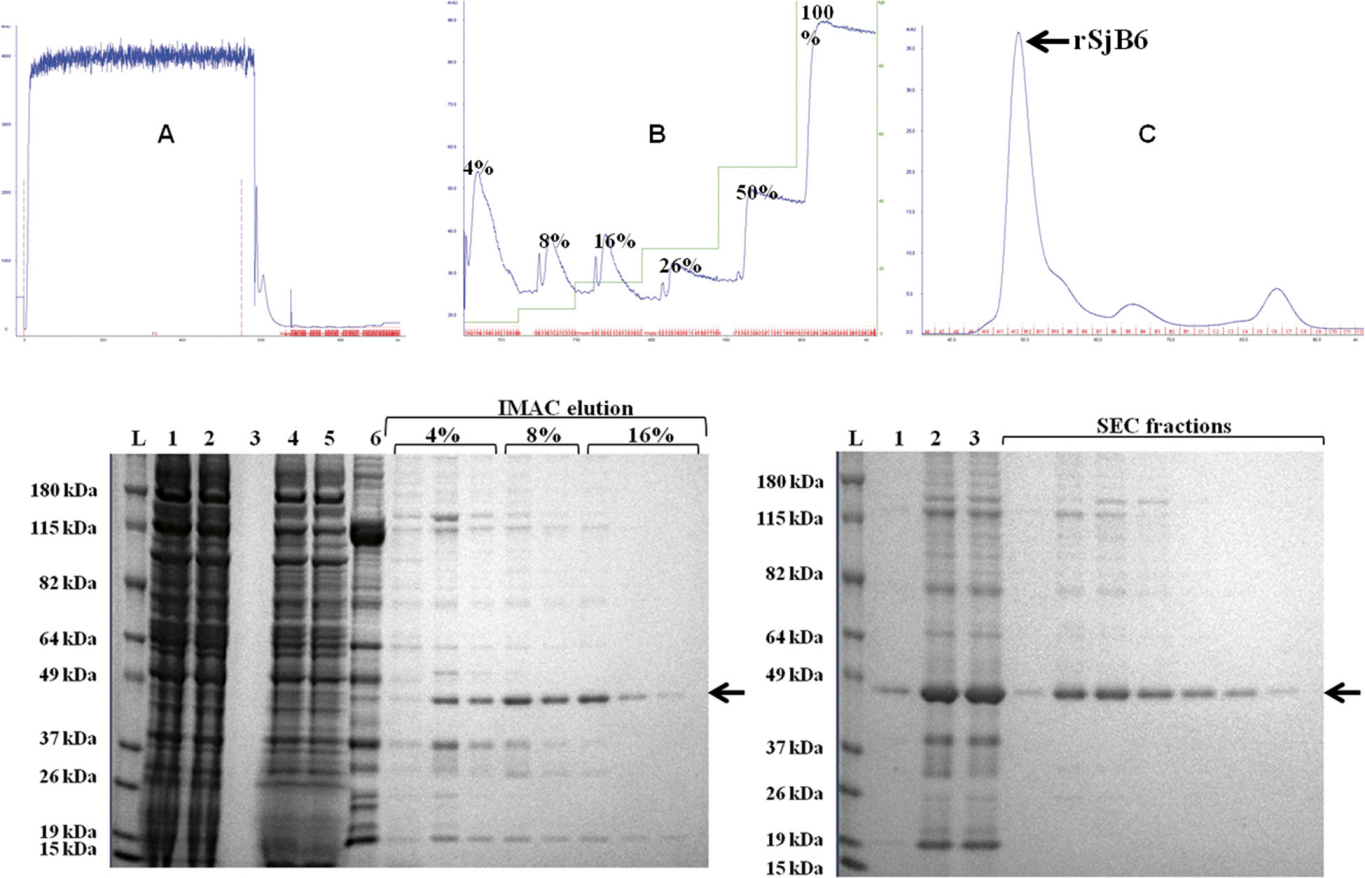
**Purification of rSjB6 expressed in Hi-5 cells by IMAC affinity chromatography followed by size exclusion chromatography.** Top panel: **(A)** An IMAC column was loaded with filtered protein sample and the column washed to remove unbound material. **(B)** Multi-step elution was carried out with increasing concentration of elution buffer (4%, 8%, 16%, 26%, 50% and 100%). The peaks indicate eluted protein fractions. **(C)** IMAC purified elution samples were pooled, concentrated and loaded onto the SEC column for further purification. The blue peak corresponds to the rSjB10 fraction. Bottom panel: Representative samples collected from the eluted fractions were analysed by SDS-PAGE. L = protein ladder, lane 1 = culture supernatant; lane 2 = filtered supernatant; lane 3 = Crossflow waste; lane 4 = concentrated supernatant; lane 5 = IMAC flow through; lane 6 = IMAC wash. Black arrows show the purified rSjB6.

The filtered supernatant was loaded into prepacked 1 ml HisTrap excel IMAC column (GE Healthcare). The column was washed with wash buffer (20 mM Sodium phosphate, 500 mM NaCl, 20 mM Imidazole, pH 7.1) before a multi-step elution, whereby increasing concentrations (4%, 8%, 16%, 26%, 50% and 100%) of elution buffer (20 mM Sodium phosphate, 500 mM NaCl, 500 mM Imidazole) were used to elute the protein of interest (Figure 1). Each concentration was held until a baseline absorbance was reached. Purification steps were performed on AKTA Explorer FPLC system (GE Healthcare) at a flow rate of 1 mL/min at 4°C. Eluted protein fractions were dialysed against phosphate buffered saline (pH 7.4) and loaded into a S75 SEC column with AKTA Explorer FPLC system at 0.5 mL/min, at 4°C. Protein fractions collected were analysed on SDS PAGE and the yield determined by standard Bradford assay [41]. The recombinant protein was stored at −20°C until needed.

### Assay of native SjB6 protein in *S. japonicum*

Production of rabbit antiserum was carried out by Antibody Production Services (South Australian Health and Medical Research Institute, Australia). Briefly, the rabbit was immunised 3 times with 250 μg rSjB6 at 3-week intervals. The polyclonal antiserum was collected 7 days after the last immunisation and stored at −80°C until required. Western blotting was performed according to the LI-COR Biosciences protocol. Briefly, rSjB6 and SWAP of *S. japonicum* and subjected to electrophoresis on a 4-12% (w/v) NuPAGE gel (Invitrogen). After separation, electrophoretic transfer of proteins from the polyacrylamide gel to Immuno-Blot LF PVDF (Bio-Rad) membrane was achieved using a XCell II Blot Module (Invitrogen) at 25 V for 2 h. Membranes were blocked with Odyssey Blocking buffer (OBT, LI-COR Biosciences) overnight at 4°C, then incubated with the primary antibodies (anti-rSjB6 antiserum and normal rabbit serum as control) for 1 h at room temperature (1:1000 dilution of rabbit antiserum in 0.2% (v/v) Tween-20 in OBT). The membranes were washed with PBST (PBS with 0.1% (v/v) Tween-20) and then incubated with secondary antibody (IRDye-labeled goat anti-rabbit IgG (H + L) at 1:20,000 dilution, LICOR Biosciences) in 0.02% (w/v) SDS in OBT for 1 h at room temperature. After further washes with PBST, the immunoreactions were visualised with a LICOR Odyssey Infrared Imager.

### Experimental challenge infection of rodents, measurement of serum anti-SjB6 antibodies

The laboratory rat is a resistant host for experimental infections with *S. japonicum* while the mouse is a susceptible host [42-44]. Two infection time-course experiments were completed with outbred Wistar rats (7 weeks old) and Swiss outbred mice (8 – 12 weeks old) (Animal Resource Centre, Australia). Rats (N = 3) and mice (N = 5) were infected percutaneously with 200 and 60 *S. japonicum* cercariae, respectively, using the cover slip method. Blood samples were collected from mice prior to infection and 6 weeks post-challenge while in the rat time-course experiment, blood samples were collected after a primary and a secondary cercarial challenge (as above) based on the observation that immune resistance in rats re-challenged with schistosomes was shown to be highest between 4–8 weeks after re-infection [45,46]. Blood was collected via the tail vein (rats), the tail tip (mice) or from cardiac puncture at necropsy. Sera were obtained from clotted blood by centrifugation and stored at −80°C until required.

The levels of anti-SjB6 antibodies in the sera of individual mice and rat were determined by standard ELISA. Briefly, microplates were coated with 0.5 μg/mL of rSjB6. Plates were washed with PBST and blocked with 5% (w/v) skim milk in PBST. After washing, plates were incubated with mouse sera in blocking buffer for a further 1.5 h at 37°C before being incubated with second antibody (goat anti-mouse IgG conjugated to HRP, Sigma Aldrich) and subsequently developed with substrate solution (SIGMAFast OPD tablets). The endpoint antibody titre was defined as the dilution with an OD reading 2 times above the background level. The protocol for ELISA using the rat sera was identical to that of the mouse except for the secondary antibody used (goat anti-rat IgG conjugated to biotin, Sigma Aldrich) and an additional incubation with HRP conjugated to streptavidin (BD Biosciences) before developing with the substrate solution. Soluble worm antigen products were used as positive control antigen.

### Statistical analysis

Data were expressed as mean ± standard error of mean (SEM). Changes in real time PCR data and immunological parameters were assessed by One-way ANOVA with post hoc Bonferroni testing (p ≤ 0.05). These analyses were performed using the GraphPad Prism version 6.02 (GraphPad Software).

## Results

### Cloning and general characteristics of *SjB6*

The cloned full-length cDNA of *SjB6* (1160 nucleotides) contained a complete open reading frame (ORF) encoding a polypeptide of 387 amino acids with a predicted molecular weight of 43.7 kDa and a pI of 6.19. Analysis of the deduced SjB6 peptide indicated the presence of three conserved motifs, namely a serpin motif, a serpin signature and a RCL (Table 1), located near the C-terminal with the cleavage site of the RCL at position 346C–S347. Three N-glycosylation sites (9NFTD12, 49NTKA52 and 209NLTS212) and one glycosaminoglycan attachment site (72SGIG75) were identified on the polypeptide. The SjB6 polypeptide contained two protein kinase C phosphorylation sites, one tyrosine phosphorylation site and microbodies C-terminal targeting signal. A signal peptide was found at the N-terminal of the polypeptide but no transmembrane domain was found indicating that SjB6 was a secretory serpin. Analysis of the SjB6 RCL showed that the hinge region contained the consensus sequence typical of the RCL hinge region, a characteristic common to all inhibitory serpins “P_17_ [E]-P_16_ [E/K/R]-P_15_ [G]-P_14_ [T/S]-P_13_ [X]-P_12–9_ [AGS]-P_8–1_ [X]-P_1′_–_4’_” [4,21], with residue variation only at position P_14_.

**Figure 2:**
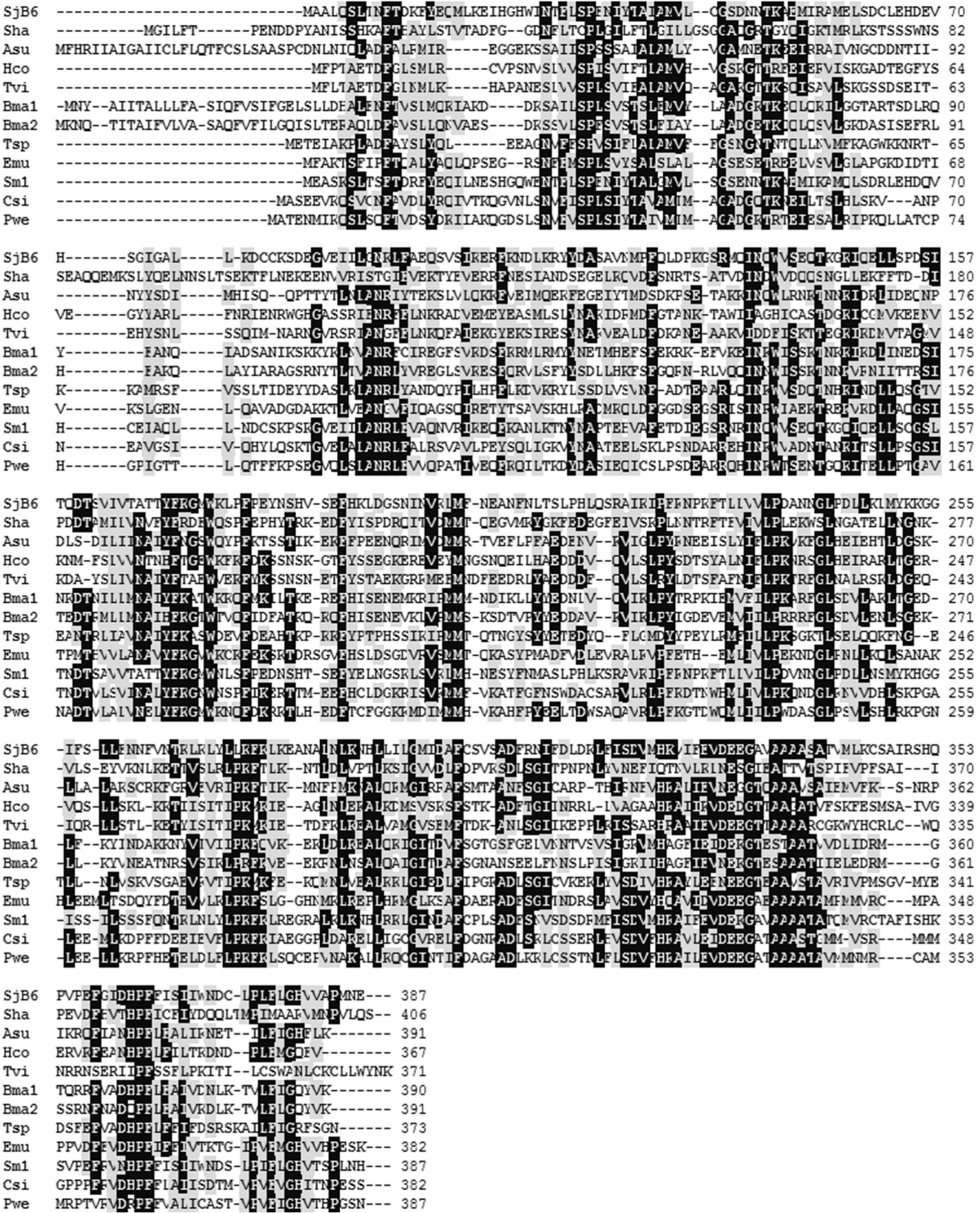
**Multiple sequence alignment comparison of the deduced amino acid sequence of SjB6 known helminth serpins.** Sha (*Schistosoma haematobium*), Emu (*Echinococcus multilocularis*), SjB6 (*S. japonicum*), Sm1 and Sm2 (*S. mansoni*), Csi (*Clonorchis sinensis*), Pwe (*Paragonimus westermani*), Hco (*Haemonchus contortus*), Tvi (*Trichostrongylus vitrinus*), Asu (*Ascaris suum*), Bma1 and Bma2 (*Brugia malayi*). All GenBank accession numbers are shown in Table 2.

**Figure 3:**
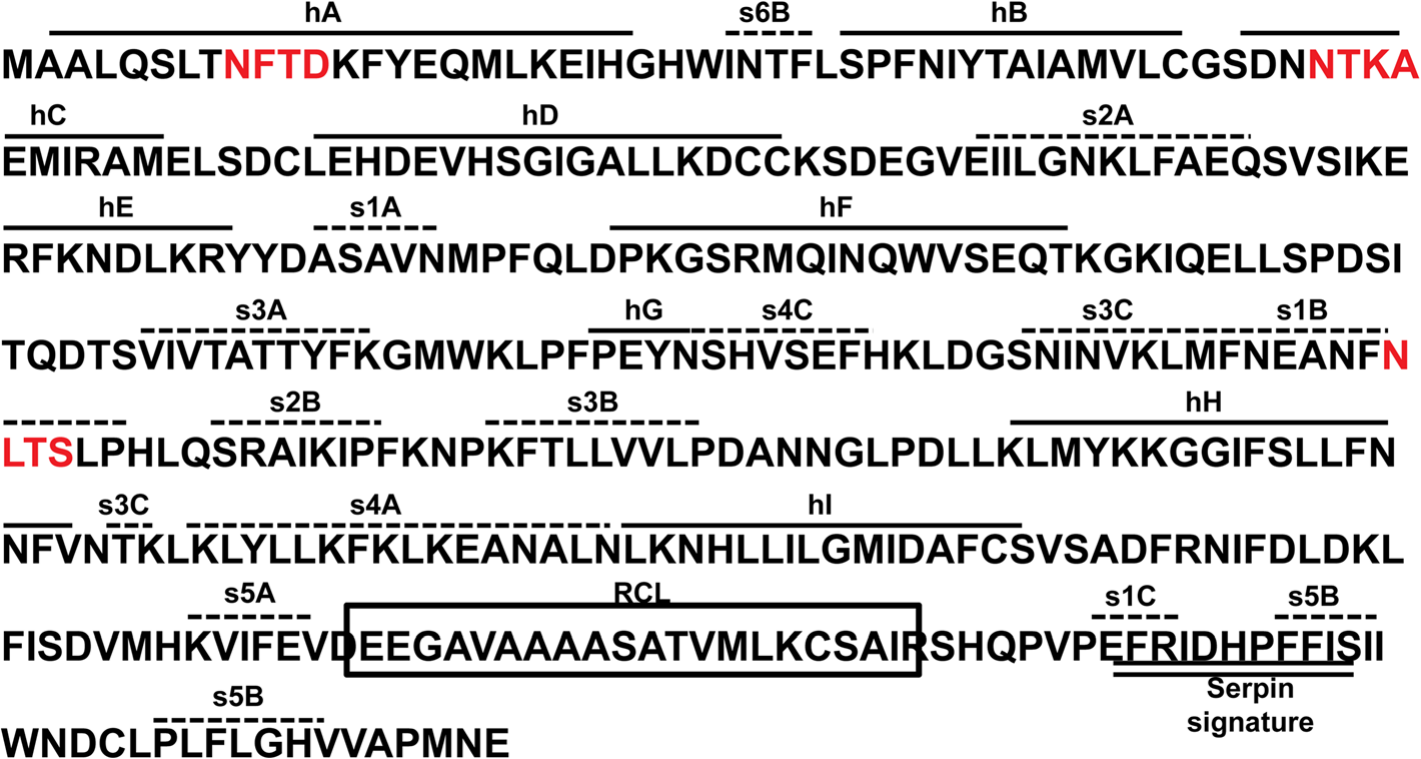
**Predicted amino acid sequence of SjB6 showing the position of conserved alpha helices and beta strands.** Secondary structures were assigned based on the 1HP7 tertiary structure; bold lines are α-helices and broken lines are β-strands. Helices are labelled from “hA” to “hI” and β-strands that constitute β-sheet A-C are labelled as “sA”, “sB” and “sC” respectively. The highly and lowly conserved residues are labelled in black and grey, respectively. Sequences highlighted in red are N-glycosylation sites. Sequences in the box represent the RCL while double bold lines represent the serpin signature.

Multiple sequence alignment of the deduced SjB6 amino acid sequence with key known parasitic helminth serpins from Genbank showed between 21 – 54% homology (Figure 2) with the highest sequence homology to *S. mansoni* serpin (Accession number CCD60349.1). SjB6 sequence showed very low overall sequence homology to serpins from other organisms including humans (data not shown). Analysis of the secondary structure using SWISS-MODEL (http://swissmodel.expasy.org/) and Phyre^2^[27] showed that SjB6 contains nine α-helices and thirteen β-strands (Figure 3).

### Structure-based alignment analysis of SjB6

**Figure 4:**
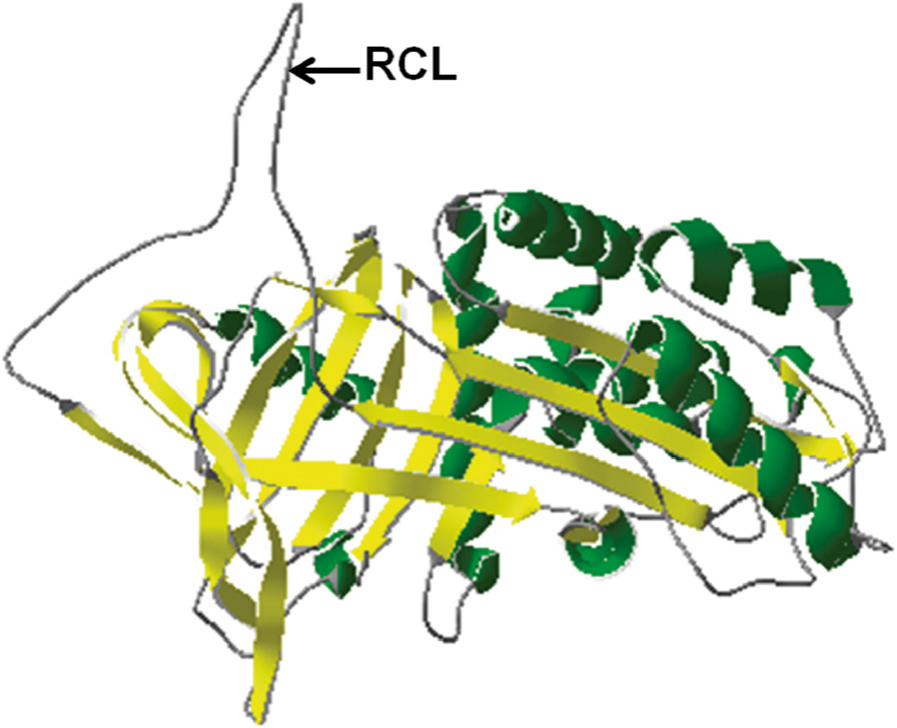
**Predicted tertiary structure of SjB6.** The figure shows the protein in its native conformation with the RCL (indicated by black arrow) being surface accessible by target protease. The green colour represents the α-helices, the yellow colour represents the β-sheets, while loops are coloured grey.

Alignment of the predicted tertiary structure of SjB6 (Figure 4) with other predicted tertiary structures of helminth serpins showed that SjB6 is structurally similar, with the greatest similarity to *S. mansoni* serpin (GenBank: CD60349.1) (Table 2). The root mean squared values ranged between 0 and 2. The root mean squared value indicates the degree of similarity between the three-dimensional structures of two proteins by measuring the root-mean-square distance between equivalent atom pairs from both proteins [47].

**Table 2:** Structural alignment of SjB6 with human α1-antitrypsin and known helminth serpins

**Serpin name**	**Genbank accession number**	**Source**	**Reference**	**RMS value (Å)**	**No of atoms involved**
α1-antitrypsin	AY256958	Humans	Bollen *et al.*[48]	1.30	288
SRP-3	AY525080	*C. elegans*	Pak *et al.*[49]	1.29	235
CsSERPIN	EF550965	*C. sinensis*	Kang *et al.*	1.15	238
Serpin^Emu^	CAD12372.2	*E. multilocularis*	Merckelbach and Ruppel [50]	1.15	235
Hc-serpin	ACP43576	*H. contortus*	Yi *et al.*[51]	1.23	247
PwSERPIN	EU014295	*P. westermani*	Hwang *et al.*[52]	1.18	239
Sh serpin	AAA19730	*S. haematobium*	Blanton *et al.*[53]	1.52	221
Sj serpin	AAK57435	*S. japonicum*	Yan *et al.*[54]	1.49	216
Smpi56	CCD60349	*S. mansoni*	Ghendler *et al.*[55]	1.16	240
Contrapsin	CCD60352	*S. mansoni*	Modha and Doenhoff [56]	1.16	240
Ts11-1	DQ864973	*T. spiralis*	Nagano *et al.*[57]	1.22	248
Tv Serp	Y12233	*T. vitrinus*	MacLennan *et al.*[58]	1.20	296
Bm-spn-1	U04206	*B. malayi*	Yenbutr and Scott [59]	1.14	305
Bm-spn-2	AF009825	*B. malayi*	Zang *et al.*[60]	1.19	298

### Phylogenetic analysis

**Figure 5:**
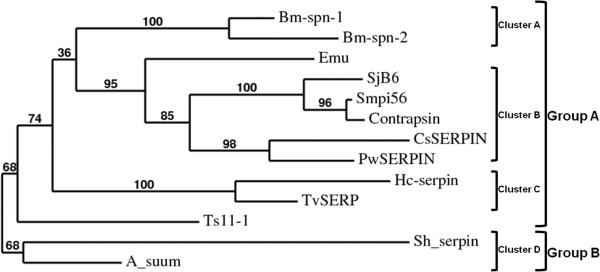
**Phylogenetic tree analysis of amino acid sequences for SjB6 and other parasitic helminth serpins.** The alignment of retrieved sequences (GenBank accession numbers shown in Table 2) was created using MUSCLE. All sequences were retrieved from GenBank.

Phylogenetic analysis of the amino acid sequence of SjB6 with other helminth parasite serpins indicated two major groups – Group A and Group B. The former comprises serpins formed by clusters A, B and C while the latter comprises serpins formed by clusters D. The 4 phylogenetic clusters were supported by the aLRT statistical test [61] showing branch support values of 100% for clusters A and C, 95% for cluster B and 65% for cluster D (Figure 5). SjB6 is closely related to Smpi56 and contrapsin grouped within cluster A and sharing 69% sequence identity.

### Gene expression profile of the *SjB6* gene

**Figure 6:**
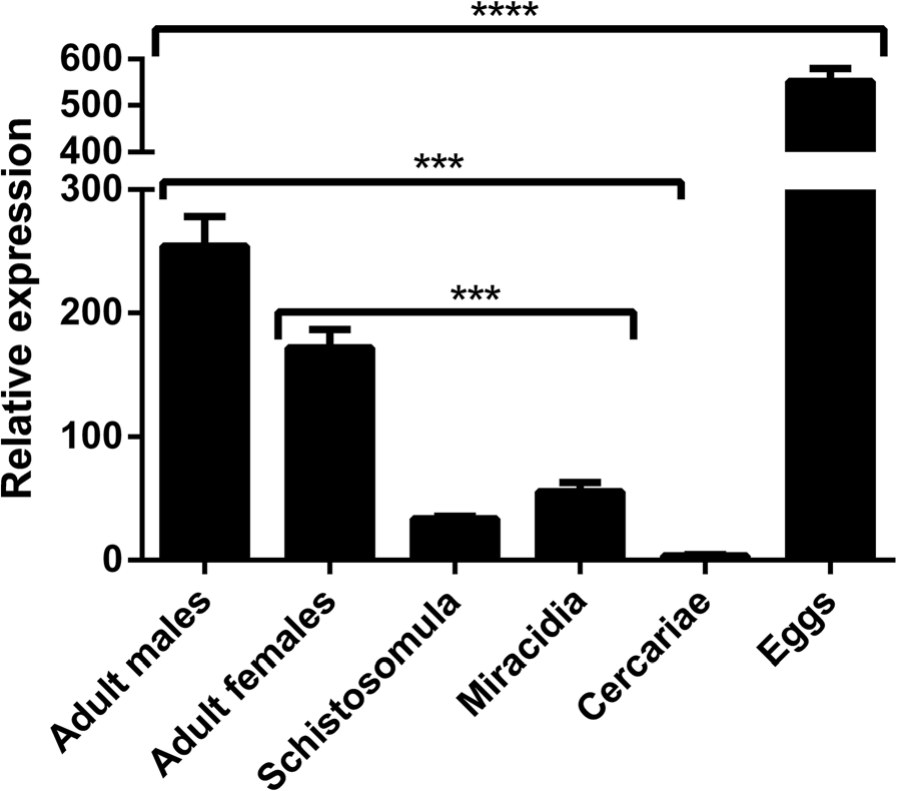
**Gene expression of *SjB6 *gene in *S. japonicum *life cycle stages.***SjB6* expression was at least 2.5-fold higher in eggs than in other life cycle stages examined. Error bars represent mean real-time PCR expression ± standard error of mean (SEM) from three biological replicates. ****p-value ≤ 0.0001, ***p-value ≤ 0.001.

Stage-specific expression of the *SjB6* transcript was examined by real-time PCR using cDNA reverse transcribed from RNA samples isolated from several developmental stages of *S. japonicum* and primers designed to specifically amplify the *SjB6* gene*.* Expression was detected only in the intra-mammalian stages of the parasite lifecycle with the egg stage showing about 3-fold increase in expression when compared with the other stages and this increase was highly significant (p ≤ 0.001) (Figure 6). The *NADH-UR* gene used as the internal control showed a constant expression across all the lifecycle stages tested (data not shown).

### Expression and purification of recombinant SjB6 *in vitro*

The recombinant SjB6 protein was expressed using the flashBAC baculovirus expression system as a secreted protein. In the first step of purification, IMAC affinity column was used to partially purify rSjB6. Further purification by size exclusion chromatography resulted in a single protein band of approximately 43 kDa as analysed on a 4-12% (w/v) NuPAGE gel (Figure 1).

### Western blot analysis of native SjB6 in *S. japonicum* adult worms

**Figure 7:**
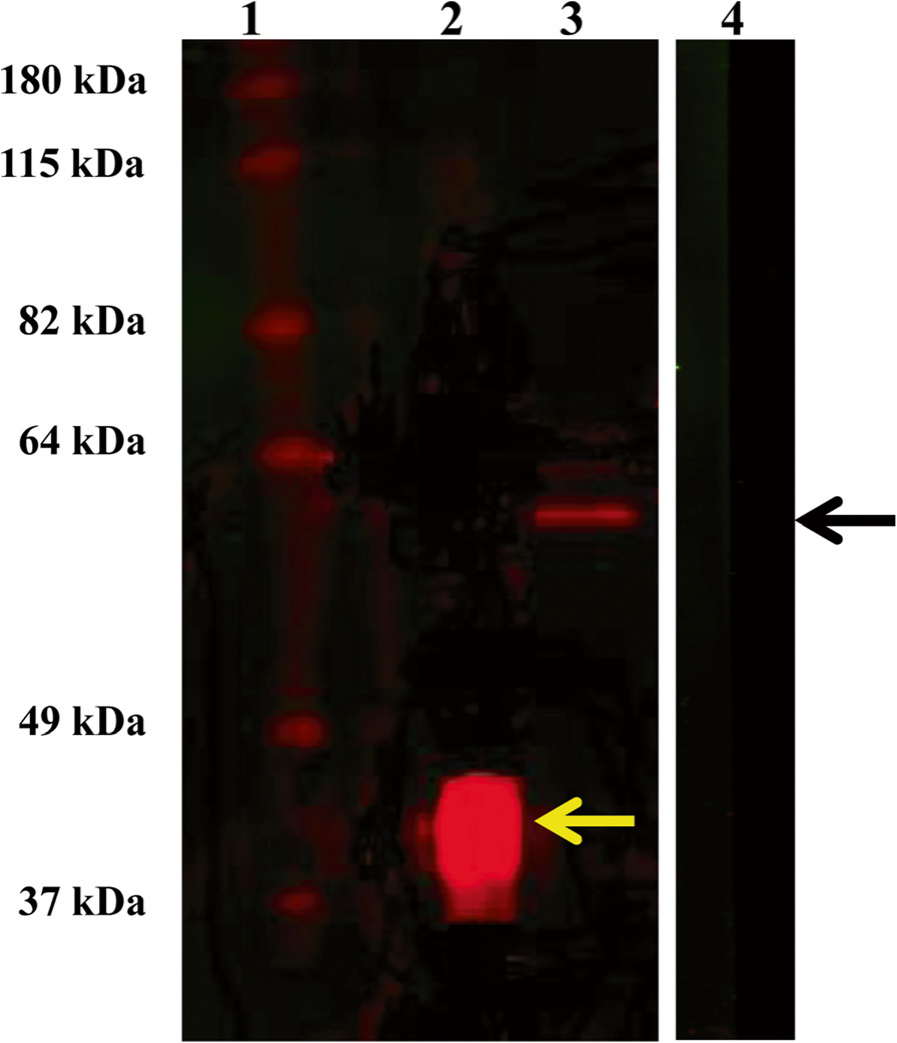
**Western blot analysis of native SjB6.** Lane 1, pre-stained protein ladder; lanes 2 and 3, rSjB6 and SWAP probed with rabbit antiserum respectively; Lane 4, SWAP probed with pre-immune rabbit serum. The positive signal at 43 kDa in lane 2 indicates the position of rSjB6 (yellow arrow) while the positive signal at 60 kDa indicates the position of native SjB6 (black arrow).

Western blotting was used to determine whether rabbit anti-serum, raised against purified rSjB6, to was able to react with the native antigen in SWAP. As shown in Figure 7 (lane 3), a highly specific positive signal at an approximate molecular weight of 60 kDa was detected in the SWAP. There was also a positive signal at the expected molecular weight of 43 kDa with the rSjB6 sample (lane 2, Figure 7), confirming the specificity of the rabbit antiserum. No signal was evident with the SWAP sample incubated with pre-immune rabbit serum (Figure 7, lane 4). The molecular weight of the protein band detected in the SWAP sample probed with rSjB6-specific rabbit antiserum was considerably higher than that of rSjB6.

### Antibody response of experimentally challenged rodents to rSjB6 and SWAP

**Figure 8:**
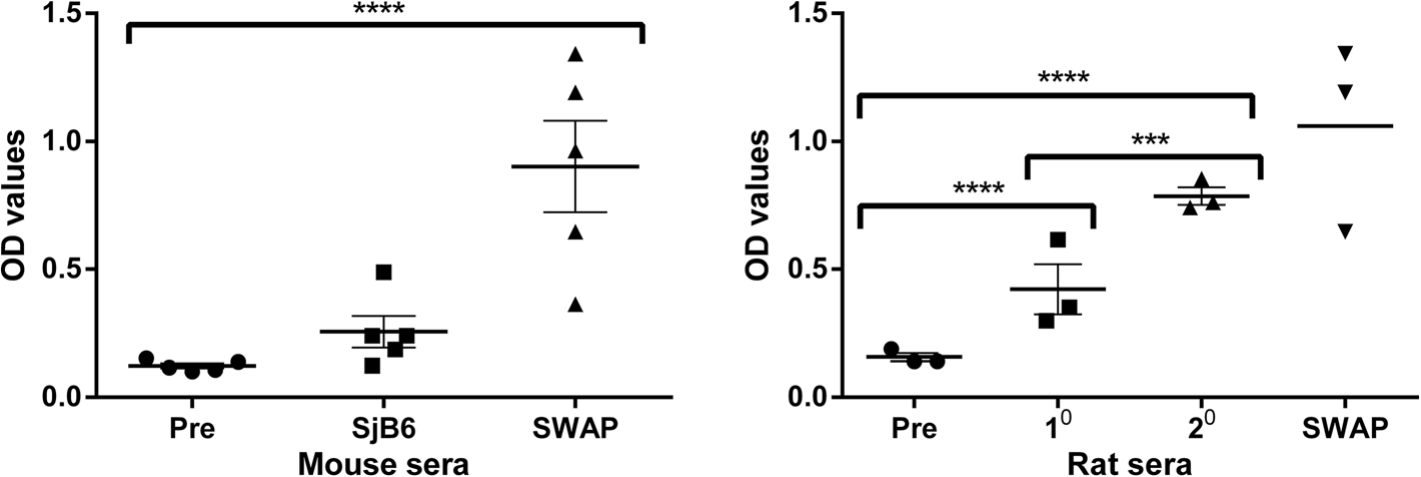
**Humoral immune profile of experimentally challenged rodents against rSjB6.** Mice (n = 5) and rats (n = 3) were experimentally challenged with *S. japonicum* cercariae and immune sera collected six weeks post infection with additional sera collected from the rats 6 weeks post-secondary challenge. Pre-immune sera were also collected prior to the cercarial challenge. Each data point represents an average of 3 replicates. There was no significant difference between the serum antibody levels in the mice before and after challenge (left panel). Rat sera showed high serum reactivity against rSjB6 after challenge (right panel). Error bars represent mean + SEM. 1° = primary challenge, 2° = secondary challenge, PRE = pre-immune sera. Statistically significant difference (Two-way ANOVA: ****= p-value < 0.0001, ***= p-value 0.0015).

An ELISA was carried to investigate the immunogenicity of SjB6 as well as the antibody response profile of experimentally challenged mice (susceptible host) and rats (resistant host) against this serpin. The ELISA results clearly showed strong rat serum antibody reactivity against rSjB6 six weeks post-infection and this response was highly significant (p < 0.0001) when compared with the rat sera obtained prior to challenge (Figure 8). The total IgG antibody titre was even more significantly higher in sera collected 6 weeks after a secondary challenge (Figure 8). These results were in direct contrast to those observed in experimentally challenged mice in which no antibody response against rSjB6 was observed six weeks post-infection (Figure 8). There was a consistently strong antibody response against SWAP in both experimentally challenged mice and rats (Figure 8) which effectively served as a positive control.

## Discussion

Although a number of serpins have been identified and characterised from many parasitic helminths [13], knowledge of schistosome serpins is limited. In this study, we describe the identification and some characteristics of a secretory serpin from *S. japonicum.*

Although the SjB6 amino acid sequence shared low overall sequence homology with other known serpins, SjB6 was identified by closer interrogation as a typical member of the serpin superfamily as it contains conserved characteristic serpin features such as the RCL, serpin signature and serpin motif [1,4,62]. In addition, the SjB6 protein is composed of 387 amino acid residues with a predicted molecular weight of 43.7 kDa and a native molecular weight of 60 kDa, which is consistent with other members of the serpin superfamily [63]. Sequence analysis demonstrated that SjB6 contains an N-terminal signal peptide with no transmembrane domain, indicative of a secretory serpin. In addition, secondary and tertiary structure prediction analysis showed that SjB6 contains 9 α-helices and 15 β-strands, features again consistent with known serpins [1,4,18,64]. RCLs of native inhibitory serpins are always exposed and accessible to target proteases [62] and this was reflected in the predicted tertiary structure of SjB6.

The consensus 20/21 residue peptide “P_17_ [E]-P_16_ [E/K/R]-P_15_ [G]-P_14_ [T/S]-P_13_ [X]-P_12–9_ [AGS]-P_8–1_ [X]-P_1’_ – _4’_” within the RCL of a serpin determines whether it is categorised as inhibitory or non-inhibitory [4,21]. As a general rule, inhibitory serpins contain glycine at position P15, threonine or serine at P14, and positions P12-P9 are occupied by alanine, glycine or serine residues with short-side chains. The corresponding regions of non-inhibitory serpins do not conform to this consensus [4]. These conserved residues of inhibitory serpins are essential for efficient and rapid insertion of the RCL into the “A” β-sheet, a process critical to inhibitory activity of a serpin [4]. Furthermore, it is also known that the presence of hydrophobic residues within the hinge region of a serpin provides an advantage for the construction of the skeleton conformation needed for the inhibitory activity of serpins [65]. Further analysis of SjB6 RCL showed that the hinge region is predominantly occupied by hydrophobic amino acid residues which further strengthen the argument that SjB6 is an inhibitory serpin. Multiple structure alignment of the predicted tertiary structure of SjB6 with other known serpins showed that SjB6 is structurally similar to helminth serpins with highest similarity to a *S. mansoni* serpin (Smpi56) with a root mean squared value of 1.16. This finding was supported by the result obtained from the phylogenetic analysis of SjB6 sequence and other parasitic helminth serpin sequences which showed that SjB6 clustered very closely with Smpi56.

Rabbit anti-rSjB6 serum detected one band (60 kDa) in native *S. japonicum* SWAP. The molecular size of the native protein was larger than the recombinantly derived polypeptide (43 kDa) but this was not surprising due to the presence of multiple N-glycosylation sites on the SjB6 polypeptide indicating that the native SjB6 undergoes post-translational modifications including glycosylation, as do many other known serpins [18,66]. However, SjB6 was undetected in the adult worm excretory/secretory (E/S) products indicating that SjB6 is not secreted as part of the E/S products (data not shown). Gene expression profiling of *SjB6* gene provided some insight into the possible biological function of SjB6 in *S. japonicum.* Real-time PCR showed that *SjB6* was expressed in the intra-mammalian stages and, especially, in eggs suggesting a possible role for SjB6 in the pathogenesis of schistosomiasis. Schistosome-induced pathology is a consequence of the host immune response against the parasites’ soluble egg antigens [67-69]. Additionally, another possible role for SjB6 is in protecting the schistosome eggs deposited in the host tissues from attack by host proteases.

Many secreted serpins have been shown to play important roles in host-parasite interactions [70-74]. We used ELISA to determine anti-SjB6 antibody levels (total IgG) in the sera of laboratory rodents experimentally infected with *S. japonicum.* The results showed that rSjB6 was strongly recognised by sera from rats experimentally infected with *S. japonicum* (Figure 8) suggesting its release into host tissue and induction of a host immune response. Surprisingly, no measurable reactivity against rSjB6 was detected in the sera of mice experimentally infected with *S. japonicum*. One possible explanation for this observation is the fact that the rat immune system is known to generally recognise more schistosome antigens than the mouse [75] and SjB6 could be one of the molecules not recognised by the murine immune system. Another possibility could be that the amount of SjB6 released by *S. japonicum* into the host may not be sufficient to induce an immune response by the mouse.

## Conclusion

The full-length cDNA encoding a secretory serpin (SjB6) from *S. japonicum* was cloned and expressed, and some of its characteristics determined, thereby providing some important insights into the biological functions of this protein. *In vitro* inhibitory and other biochemical characterisation as well as *in vivo* studies, gene knock-down experiment transgenesis are required to further our understanding of the biology of SjB6 and its possible roles in the host-parasite interaction.

## Competing interests

The authors declare that they have no competing interest.

## Authors’ contributions

AJM, GNG and DPM conceived and designed the study. AJM performed all experiments except the experimental challenge of animals carried out by PD. AJM analysed all experimental data and wrote the entire manuscript. Manuscript was drafted by AJM, GND and DPM. All authors approved the final version of manuscript.
